# Exploring the interplay between tinder use, general self-esteem, and sexual self-esteem among European emerging adults

**DOI:** 10.1186/s40359-026-04599-y

**Published:** 2026-04-29

**Authors:** Jalisse Schmid, Marco Lauriola, Christina Stadler, Eva Unternaehrer

**Affiliations:** 1 Child- and Adolescent Psychiatric Research Department, University Psychiatric Clinics Basel (UPK), Basel, Switzerland; 2 Department of Developmental and Socialization Processes, Sapienza University of Rome, Rome, Italy

**Keywords:** Tinder, Online dating, General self-esteem, Sexual self-esteem

## Abstract

**Background:**

The use of online dating apps such as Tinder has become widespread among emerging adults. However, findings regarding the impact of Tinder use on users’ self-esteem are mixed, and it remains unclear whether associations differ across general and sexual self-esteem domains. The present study examined whether Tinder use and its frequency were associated with general and sexual self-esteem in a large sample of emerging adults from Switzerland and Italy.

**Methods:**

A total of 2,283 participants (*M*_*age*_ = 23.9 ± 3.0, 76.3% female, 60.3% Italian) completed an anonymous online survey assessing Tinder use (former/current or never), frequency of use, and standardised measures of general self-esteem (Rosenberg Self-Esteem Scale) and sexual self-esteem (Sexuality Scale). We conducted four multiple linear regression analyses to test whether Tinder use or Tinder use frequency among users predicted general and sexual self-esteem, while controlling for sex, age, socioeconomic status, education, relationship status, and nationality.

**Results:**

Tinder use was common (64.7% former or current users), and Tinder users reported slightly lower general self-esteem than non-users (*p* = .044), whereas use frequency showed no association with self-esteem. In contrast, Tinder use was positively associated with sexual self-esteem (*p* < .001), whereas use frequency showed no significant association among users.

**Conclusions:**

Tinder use was associated with lower general self-esteem and higher sexual self-esteem, whereas frequency of use among users showed no association in either domain. These findings highlight distinct and divergent pathways between online dating behaviour and self-perceptions, underscoring the importance of considering both general and sexual self-esteem when examining the psychological correlates of dating app use.

**Supplementary Information:**

The online version contains supplementary material available at 10.1186/s40359-026-04599-y.

## Introduction

Tinder is one of the most widely used dating apps worldwide, with over 10 million daily active users [6]. Tinder is particularly popular among emerging adults, with users’ ages generally ranging from late adolescence to the early thirties [20]. Users create profiles including photos, short descriptions, and preferences for potential partners’ gender, age, and proximity. Tinder uses gamification elements such as the swiping mechanic, where users swipe right to express interest or left to pass, creating rapid, game-like feedback loops in partner selection [22]. This design places particular emphasis on appearance-based judgements, as decisions are made primarily on the basis of profile photographs. When two users swipe right on each other, they can begin a conversation [39].

Although Tinder has often been portrayed as a superficial “hook-up app,” research indicates a wide range of user motivations beyond casual sex [12, 37, 44]. According to the Uses and Gratifications Theory, individuals choose media that satisfy specific psychological, social, or sexual needs [13]. Users report motivations such as entertainment, boosting self-esteem, curiosity, passing time, social interaction, and romantic connection [24, 39, 43, 45]. Sumter et al. [39] identified six primary motivations for Tinder use (Love, Casual Sex, Self-Worth Validation, Thrill of Excitement, Ease of Communication, and Trendiness) and found that the desire for romantic connection was often stronger than the desire for casual sex among emerging adults. Demographic factors also shape dating app motivations: women are more likely to use dating apps to find committed love, friendships, or self-esteem enhancement, whereas men more often report casual sex as a primary motivation [1, 5, 24, 27, 39]. Age also appears relevant, with older emerging adults more likely to report both love- and sex-driven motivations [39].

General self-esteem, understood as a positive or negative evaluation of one’s self-worth [31], is closely linked to psychological functioning. Higher general self-esteem is associated with greater happiness, life satisfaction, and better physical and mental health [9, 11, 32, 51], as well as more open communication, including discussions about sexual satisfaction and safe sex with partners [18, 40]. Lower general self-esteem, conversely, is associated with depression, anxiety, and other adverse outcomes [15, 35, 36].

Despite Tinder’s potential to facilitate new social and romantic opportunities, concerns have been raised about its impact on users’ self-perceptions. Some evidence suggests that compared to non-users, Tinder users report more negative body image, and male users in particular report lower self-esteem [38]. The app’s focus on appearance and the experience of repeated evaluation may negatively affect psychological well-being [24]. While some users may experience temporary boosts from receiving matches, reinforcing the app as a source of external affirmation [39], recent evidence suggests that problematic Tinder use relates primarily to users’ motives, emotional needs, and in-app experiences, rather than to global differences in general well-being [45]. Beyond use status (yes/no), frequency of use may be particularly relevant, as more intensive engagement with technology has been associated with greater exposure to its potential psychological effects [14, 42]. Examining frequency alongside use status therefore allows for a more nuanced understanding of dose–response patterns.

Importantly, recent research emphasises the need to distinguish general self-esteem from domain-specific forms of self-esteem [8, 10, 25, 46]. A person may have high general self-esteem yet feel insecure in sexual contexts, and global measures may therefore overlook important self-perception differences, especially in intimate domains [21]. Sexual self-esteem, commonly defined as confidence, positive regard, and a sense of value regarding one’s sexuality, captures how attractive, competent, and comfortable individuals feel as sexual beings. It reflects perceived sexual acceptability, sexual identity, and confidence in managing sexual thoughts, emotions, and behaviours [29, 50]. Higher sexual self-esteem is associated with healthier sexual functioning, greater sexual satisfaction, and safer sexual practices, and it is an important predictor of intimacy-related behaviours such as more open and honest sexual communication [23].

The increasing use of dating apps raises new questions about sexual self-esteem. Repeated exposure to evaluations of one’s desirability may either boost or undermine confidence as a sexual partner [4, 41]. Existing findings are mixed: some studies show that Tinder users, especially men, report more positive sexual attitudes and higher sexual confidence [3, 33], whereas others report greater sexual dissatisfaction and sexual worry [2]. Still other work finds no association between Tinder use and sexual self-esteem [45]. Notably, most studies did not distinguish between general and sexual self-esteem, despite evidence that these constructs behave differently and may show divergent associations with sexual and online behaviours [17, 19, 21, 30].

Together, these findings suggest that dating apps such as Tinder could act as a “double-edged sword” for self-perceptions. On the one hand, swiping-based environments may provide opportunities for connection, feedback, and sexual exploration; on the other hand, they may intensify appearance-based comparison, objectification, and reliance on external validation. From this perspective, general and sexual self-esteem may be differentially sensitive to online dating experiences, with global self-worth remaining relatively stable while more domain-specific evaluations of one’s sexual self may be more vulnerable to repeated partner selection and rejection in app environments.

### Present study

The question of how individuals evaluate themselves as sexual partners has gained increasing relevance in the context of dating apps, where appearance-based judgments and rapid feedback are central features. Because Tinder is widely used among emerging adults and emphasises visual self-presentation, swiping-based selection, and sexualised interactions, it may meaningfully shape both general and sexual self-esteem. Yet, despite theoretical arguments and mixed empirical findings, the relationship between Tinder use and different forms of self-esteem remains unclear. The present study examined whether Tinder engagement (both use (yes/no) and frequency of use among users) was associated with general and sexual self-esteem in a large sample of emerging adults. To our knowledge, this is one of the first studies to simultaneously examine both constructs within the same sample, allowing for a direct comparison of their associations with Tinder use.

We included sex, age, socioeconomic status, level of education, relationship status, and nationality (Italian versus Swiss) as covariates, as these factors are known to be associated with both app engagement and self-esteem outcomes [1, 2, 6, 24, 38, 45]. For example, higher self-esteem tends to be associated with male sex, higher socioeconomic status, higher educational attainment, and being in a relationship [2, 6, 25, 35, 39, 45]. Controlling for these covariates allowed us to isolate the unique associations of Tinder use with each self-esteem outcome. Because general and sexual self-esteem are moderately correlated yet conceptually distinct constructs [21], each outcome was also adjusted for the other; not to imply a causal relationship between them, but to partial out their shared variance and obtain estimates of Tinder use that are specific to each domain.

We hypothesised that (H1a) general self-esteem would differ between Tinder users and non-users after controlling for sexual self-esteem and sociodemographic covariates, and (H1b) Tinder use frequency would be associated with general self-esteem when the same covariates were included. Furthermore, we expected that (H2a) sexual self-esteem would differ between Tinder users and non-users after controlling for general self-esteem and sociodemographic covariates, and (H2b) Tinder use frequency would be associated with sexual self-esteem when the same covariates were included. In addition to these primary hypotheses, we conducted exploratory analyses examining associations between Tinder use motivations and both general and sexual self-esteem. Although motivations were not a primary focus of this study and no formal hypotheses were specified, their inclusion was theoretically motivated by evidence suggesting that why individuals use Tinder may matter more than whether or how often they use it [45].

## Methods

### Participants

A total of 3,655 individuals were recruited to participate in this online study. Undergraduate students enrolled in psychology courses at the University of Basel were invited to participate via the Bapsona research participation system and received course credit (*N* = 487, 21.3% of the final sample). The remainder of the sample was recruited through leafleting and social media (Instagram, Facebook, WhatsApp, LinkedIn), targeting emerging adults across Switzerland and Italy. Inclusion criteria were: (1) being between 18 and 30 years old (*N* = 369 excluded), (2) being Italian or Swiss, or residing in Italy or Switzerland (*N* = 75 excluded), and (3) having completed the full online survey (*N* = 923 excluded). In addition, five participants who identified as non-binary were excluded from analyses because of insufficient group size (*N* = 5 excluded). The final sample consisted of 2,283 emerging adults.

The final sample had a mean age of 23.9 (*SD* = 3.0) years and was predominantly female (76.3%). Swiss participants represented 39.7% and Italians 60.3%. For further descriptive information, please refer to Table 1.

**Table 1:** Participant characteristics and group differences between Tinder users and non-users (*N* = 2,283)

	Tinder Users			Non-Tinder Users			Difference	
Variable	*N*	*M*	*SD*	*N*	*M*	*SD*	*estimate*	*p-value*
Age (years)	1476	24.3	3.0	807	23.1	2.9	*t*(1700.6) = −9.18 *d* = −0.40	<.001
Self-esteem score	1476	18.0	5.6	807	18.4	5.2	*t*(1749.8) = 2.02 *d* = 0.09	0.043
Sexual self-esteem score	1476	3.3	9.1	807	2.5	8.8	*t*(1657.2) = −2.22 *d* = −0.10	0.027
Gender identification:^1^ woman	1078	4.5	0.8	650	4.6	0.7	W = 333,605	0.048
Gender identification:^1^ man	382	4.4	0.8	152	4.5	0.8	W = 27,236	0.205
		*N*	*%*		*N*	*%*		
Biological sex assigned at birth							χ^2^(1) = 14.304	<.001
Female		1089	73.8		653	80.9		
Male		387	26.2		154	19.1		
Education							χ^2^(1) = 40.31	<.001
Up to high school degree^2^		635	43.5		458	57.5		
University degree^3^		826	56.5		338	42.5		
Nationality							χ^2^(1) = 132.98	<.001
Switzerland		457	31.0		450	55.8		
Italy		1019	69.0		357	44.2		
Employment^4^								
Yes, part time student		76	5.1		47	5.8	χ^2^(1) = 0.34	0.558
No, job seeker		126	8.5		63	7.8	χ^2^(1) = 0.28	0.599
Yes, part time job		311	21.1		179	22.2	χ^2^(1) = 0.32	0.572
Yes, full time job		396	26.8		147	18.2	χ^2^(1) = 20.88	<.001
Yes, full time study		732	49.6		497	61.6	χ^2^(1) = 29.71	<.001
No answer		34	2.3		13	1.6	χ^2^(1) = 0.92	0.337
Socioeconomic status							W = 553,203	0.051
Possible to live well with current income		781	55.9		458	60.4		
Current income is just enough		457	32.7		222	29.3		
It is difficult to make ends meet		159	11.4		78	10.3		
Relationship status							χ^2^(1) = 114.36	<.001
No		931	64.3		326	40.8		
Yes		517	35.7		473	59.2		
Sexual orientation							χ^2^(4) = 26.417	<.001
Heterosexual		1097	74.3		650	80.5		
Homosexual		58	3.9		12	1.5		
Bisexual		246	16.7		98	12.1		
Asexual		4	0.3		9	1.1		
No answer/not specified/not sure		71	4.8		38	4.7		

This table summarises the sociodemographic characteristics of the full sample and compares Tinder users with non-users

*Abbreviations*: *N* sample size, *M* mean, *SD* standard deviation, *t* t-statistic, *d* Cohen’s d (effect size), *W* Wilcoxon rank-sum statistic, *χ*^*2*^ chi-square statistic,* p p*-value

^1^Gender identification was assessed separately for participants assigned female and male at birth. Participants assigned female at birth rated how strongly they identify as a woman (0 = “no identification” to 5 = “strong identification”), and participants assigned male at birth rated how strongly they identify as a man on the same scale. Higher scores indicate stronger identification with one’s respective gender category. Biological sex assigned at birth (female, male) was measured as a separate variable

^2^“Up to high school degree” includes: No school diploma, Completed compulsory education (lower secondary school, elementary school), Vocational apprenticeship with Eidgenössisches Fähigkeitszeugnis (EFZ), Vocational apprenticeship with vocational baccalaureate (BM), Diploma secondary school (e.g., WMS, FMS, IMS), and Matura/Gymnasium (Secondary II)/Abitur

^3^“University degree” includes Bachelor’s, Master’s, PhD, and equivalent university-level qualifications

^4^Employment: multiple answers possible

### Procedure

The recruitment period was active from October 2022 to May 2025. The anonymous online survey was administered in German, Italian, French, and English via Qualtrics XM (Qualtrics, Provo, UT, USA). The first page of the survey contained information about the purpose of the study and participants’ rights. Participants were assured of anonymity and reminded that they could withdraw at any time. At the end of the first page, they were asked whether they wished to participate (yes/no). Only respondents between 18 and 30 years of age who gave active consent by clicking “Yes” continued with the survey; those who declined were automatically redirected to the end of the survey. Completion took approximately 10–15 min. Psychology students at the University of Basel received course credit for participation; no other form of compensation was provided to participants recruited through other avenues.

### Measures

#### Demographic information

The survey assessed age, sex, gender, nationality, citizenship, socioeconomic status (household income situation), highest level of education, years of education, working/studying situation, romantic relationship status, civil status, and sexual orientation. Self-perceived socioeconomic status (SES) was originally assessed with three categories (“with the current income it is possible to live well,” “the current income is just enough,” and “with the current income it is difficult to make ends meet”). For regression analyses, the latter two categories were combined to create a binary SES variable (0 = lower SES, 1 = higher SES) to ensure sufficient group sizes and facilitate interpretation. Sex was measured as sex assigned at birth (female or male). Gender was assessed on two separate 6‑point Likert items (0 = not at all, 5 = very strongly) asking “How strongly do you feel you belong to the female gender?” and “How strongly do you feel you belong to the male gender?”.

#### Tinder use

Participants were asked whether they were currently using Tinder or had ever used Tinder in their lifetime (yes/no response option). Users were then asked to specify whether they were current or former users, and to indicate the frequency of their Tinder use (1 = once or twice, 2 = monthly, 3 = 1–3 times a week, 4 = 4–5 times a week, 5 = every day). Non-users were not asked any follow-up questions about Tinder use.

#### Tinder use outcomes

Tinder users were additionally asked whether they had ever met a Tinder match offline for a date (yes/no) and how many one-night stands they had had with Tinder matches. These items were included to provide descriptive context for the nature of participants’ Tinder experiences, consistent with prior work characterising dating app user samples [39], and are reported in Table 2. These variables were not part of the primary hypotheses.

**Table 2:** Descriptive statistics of Tinder use, offline dating, one-night stands, and main Tinder motivations among Tinder users (*N* = 1,476); valid N varies by item

Variable	*N*	*%*	
Tinder use			
Current	438	32.2	
Not anymore	922	67.8	
Offline dates			
Female	859	62.5	
Male	233	17.0	
One-night stands^1^			
Female	495	47.6	
Male	134	40.1	
Tinder frequency			
Once or twice	240	17.5	
Monthly	177	12.9	
1–3 times a week	404	29.3	
4–5 times a week	288	21.0	
Every day	265	19.3	
Motivations^2^	*N*	*M*	*SD*
1. It is an easy way to meet someone	1455	4.0	1.0
2. To pass the time, especially when I’m bored	1461	3.9	1.0
3. To flirt	1453	3.7	1.1
4. It is fun	1462	3.7	1.0
5. I think it is funny	1461	3.7	1.1
6. I enjoy browsing on Tinder	1458	3.7	1.1
7. When I have nothing better to do	1449	3.6	1.2
8. It can be exhilarating	1452	3.6	1.1
9. It is entertaining	1452	3.5	1.1
10. To contact a possible future romantic partner	1445	3.4	1.2

This table reports descriptive statistics for participants who used Tinder, including whether they were current or former users, their offline dating and one-night stand experiences, frequency of Tinder use, and the ten highest-rated Tinder motivations. These variables provide an overview of how participants engaged with the app and their reasons for using it

*Abbreviations*:* N* sample size, *%* percentage, *M* mean, *SD* standard deviation

^1^*N* = Number of Tinder users who reported at least one one-night stand; *%* = Percentage of Tinder users with a valid response who reported at least one one-night stand (based on 1,040 female and 334 male respondents). ^2^Motivations = Participants rated 46 possible motivations for using Tinder on a 5-point Likert scale (1 = completely disagree to 5 = completely agree). The 10 motivations with the highest mean endorsement ratings are displayed in this table. Responses marked as ‘Not Applicable’ were recoded as missing and excluded pairwise from analyses

#### Tinder motivations

The Tinder Motivations Scale [39] consisted of 46 items rated on a 5-point Likert scale (1 = completely disagree to 5 = completely agree), covering motivations such as seeking love (e.g., “to find someone to be with”), casual sex (e.g., “to find someone to have sex with”), and self-worth validation (e.g., “to feel more attractive”). The scale has been validated in English and German [39].^1^ A “Not Applicable” response option was added to allow participants to distinguish between motivations they actively disagreed with and those they considered entirely irrelevant to their Tinder experience; such responses were treated as missing in analyses.^2^

#### General self-esteem scale

The Rosenberg Self-Esteem Scale (RSES; [28]) is one of the most widely used measures of general self-esteem in the social sciences [21]. It is a unidimensional scale assessing general feelings of self-worth, self-competence, and self-acceptance across 10 items (e.g., “On the whole, I am satisfied with myself”). Items were rated on a 4-point Likert scale (1 = strongly agree to 4 = strongly disagree), with five items reverse-scored. For scoring, responses were recoded to a 0–3 range, yielding possible summed scores from 0 to 30, with higher scores indicating higher general self-esteem. The scale has been validated in German, Italian, French, and English. In the present study, internal consistency was high (Cronbach’s α = 0.88). A “Not Applicable” response option was included; such responses were treated as missing.^3^

#### Sexual self-esteem scale

We used the sexual self-esteem subscale of the Sexuality Scale [34], a 10-item measure assessing confidence, positive regard, and a sense of value regarding one’s sexuality (e.g., “I am a good sexual partner”). Items are rated on a 5-point Likert scale (ranging from + 2 = strongly agree to −2 = totally disagree), with five items reverse-scored. Possible summed scores range from −20 to + 20, with higher scores indicating higher sexual self-esteem. The scale was originally validated in English.^4^ In the present study, internal consistency was excellent (Cronbach’s α = 0.94). A “Not Applicable” response option was included and treated as missing, consistent with the approach used for the other scales.^3^

### Data analysis

Data were processed and analysed using R version 4.4.2 [26] and RStudio version 2024.04.0, using the packages “haven” [49], “tidyverse” [48], and “summarytools” [7]. Graphical illustrations were created using ggplot2 version 3.5.1 [47].

To test our four hypotheses, we estimated a separate multiple linear regression model for each, predicting general self-esteem or sexual self-esteem from either Tinder use (coded 0 = never used, 1 = former or current user) or Tinder use frequency (coded on a 5-point ordinal scale: 1 = once or twice, 2 = monthly, 3 = 1–3 times a week, 4 = 4–5 times a week, 5 = every day; only Tinder users contributed to these models). All models were adjusted for sex assigned at birth, age, socioeconomic status, education, relationship status, and nationality. Models predicting general self-esteem were additionally adjusted for sexual self-esteem, and models predicting sexual self-esteem were additionally adjusted for general self-esteem. Both unadjusted and adjusted model results are presented in Table 3; only adjusted results are discussed in-text.

**Table 3:** Multiple linear regression models predicting general and sexual self-esteem from Tinder use, Tinder use frequency, and covariates

	***B***	***SE(B)***	***β***	***t***	***p***
H1a. General self-esteem predicted by Tinder use (yes/no)					
Unadjusted Model (*N* = 2080; *Adj. R*^2^ =.002)					
(Constant)	18.567	0.200	-	92.750	<.001
Tinder use (no = 0, yes = 1)	−0.569	0.248	−0.050	−2.291	0.022
Full Model (*N* = 2080; *Adj. R*^*2*^ = 0.198)					
(Constant)	17.267	1.008	-	17.136	<.001
Tinder use (no = 0, yes = 1)	−0.477	0.237	−0.042	−2.011	0.044
Sexual self-esteem	0.221	0.013	0.368	16.521	<.001
Sex (ref = female)	0.594	0.245	0.046	2.424	0.015
Age (years)	−0.002	0.046	−0.001	−0.041	0.968
Socioeconomic status (ref = high)	−1.364	0.219	−0.125	−6.232	<.001
Education (ref = no university degree)	1.007	0.270	0.093	3.721	<.001
Relationship status (ref = no relationship)	−0.450	0.227	−0.041	−1.977	0.048
Nationality (ref = Italians)	1.831	0.226	0.166	8.086	<.001

	*B*	*SE(B)*	*β*	*t*	*p*
H1b. General self-esteem predicted by Tinder use frequency					
Unadjusted Model (*N* = 1261; *Adj. R*^*2*^ =.001)					
(Constant)	18.035	0.402	-	44.913	<.001
Tinder use frequency	−0.032	0.118	−0.008	−0.275	0.783
Full Model (*N* = 1261; *Adj. R*^*2*^ =.192)					
(Constant)	16.337	1.309	-	12.484	<.001
Tinder use frequency	−0.031	0.110	−0.008	−0.287	0.774
Sexual self-esteem	0.228	0.017	0.375	13.149	<.001
Sex (ref = female)	0.646	0.324	0.049	1.996	0.046
Age (years)	0.020	0.057	0.011	0.359	0.720
Socioeconomic status (ref = high)	−1.274	0.292	−0.114	−4.364	<.001
Education (ref = no university degree)	0.714	0.351	0.064	2.037	0.042
Relationship status (ref = no relationship)	−0.382	0.301	−0.033	−1.269	0.205
Nationality (ref = Italians)	2.068	0.313	0.172	6.610	<.001

	*B*	*SE(B)*	*β*	*t*	*p*
H2a. Sexual self-esteem predicted by Tinder use (yes/no)					
Unadjusted Model (*N* = 2080; *Adj. R*^2^ =.001)					
(Constant)	2.520	0.333	-	7.559	<.001
Tinder use (no = 0, yes = 1)	0.742	0.413	0.039	1.795	0.073
Full Model (*N* = 2080; *Adj. R*^*2*^ =.190)					
(Constant)	−16.840	1.784	-	−9.438	<.001
Tinder use (no = 0, yes = 1)	1.889	0.395	0.100	4.778	<.001
General self-esteem	0.618	0.036	0.372	17.236	<.001
Sex (ref = female)	−0.253	0.425	−0.012	−0.596	0.551
Age (years)	0.266	0.074	0.089	3.567	<.001
Socioeconomic status (ref = high)	−0.076	0.364	−0.004	−0.209	0.834
Education (ref = no university degree)	−1.071	0.438	−0.059	−2.446	0.014
Relationship status (ref = no relationship)	3.561	0.366	0.197	9.735	<.001
Nationality (ref = Italians)	0.197	0.398	0.011	0.495	0.621

	*B*	*SE(B)*	*β*	*t*	*p*
H2b. Sexual self-esteem predicted by Tinder use frequency					
Unadjusted Model (*N* = 1261; *Adj. R*^*2*^ =.000)					
(Constant)	3.259	0.660	-	4.937	<.001
Tinder use frequency	−0.043	0.194	−0.006	−0.224	0.823
Full Model (*N* = 1261; *Adj. R*^*2*^ =.174)					
(Constant)	−14.751	2.270	-	−6.498	<.001
Tinder use frequency	−0.048	0.179	−0.007	−0.267	0.789
General self-esteem	0.630	0.046	0.383	13.746	<.001
Sex (ref = female)	−0.931	0.540	−0.043	−1.725	0.085
Age (years)	0.259	0.092	0.086	2.821	0.005
Socioeconomic status (ref = high)	0.494	0.482	0.027	1.024	0.306
Education (ref = no university degree)	−0.954	0.559	−0.052	−1.706	0.088
Relationship status (ref = no relationship)	2.635	0.487	0.139	5.410	<.001
Nationality (ref = Italians)	0.077	0.548	0.004	0.140	0.889

This table presents four multiple linear regression models predicting general and sexual self-esteem from Tinder use (yes/no), Tinder use frequency, and sociodemographic covariates. Models predicting general self-esteem additionally adjust for sexual self-esteem, and vice versa. Due to heteroscedasticity, bootstrapped standard errors (2,000 resamples) were used

Unadjusted Model = simple linear regression with predictor only. Full Model = multiple regression including covariates. Reported R^2^ values are adjusted R^2^. Reference categories (comparison direction): Tinder use = non-users (0) compared with users (1); sex = females compared with males; socioeconomic status = high compared with low; education = no university degree compared with university degree; relationship status = not in a relationship compared with in a relationship; nationality = Italians compared with Swiss. All coefficients represent the difference in general (or sexual) self-esteem relative to the corresponding reference group. Variables treated as continuous (Tinder frequency, age, general self-esteem, sexual self-esteem) do not have reference categories

Sample sizes vary across models due to missing data on covariates; full models were estimated using listwise deletion

*Abbreviations*: *B* unstandardised coefficient, *SE(B)* standard error of B, *β* standardised beta coefficient, *t* t-statistic, *p p*-value, *N* sample size, *Adj. R*^*2*^ adjusted R-squared, *ref* reference group

Model assumptions of linearity, normality, homoscedasticity, and multicollinearity were examined prior to interpretation. Breusch-Pagan tests indicated significant heteroscedasticity in all four models (*p* < 0.001); bootstrapped standard errors (2,000 resamples) were therefore applied to obtain robust parameter estimates. Both unstandardised and standardised coefficients are reported in Table 3. All tests were two-tailed with an alpha level of 0.05.^5^

## Results

### Descriptive statistics regarding Tinder use

Details on participants’ characteristics are reported in Table 1. Table 2 presents an overview of the Tinder users’ characteristics. Among Tinder users, 32.2% were current users and 67.8% were former users; 73.8% identified as female and 26.2% as male. The most frequently reported usage was 1 to 3 times per week (29.4%). Participants reported diverse motivations for using Tinder, spanning all six subscales of the Tinder Motivations Scale [39]: Love, Casual Sex, Ease of Communication, Self-Worth Validation, Thrill of Excitement, and Trendiness.

### Inferential analyses

#### H1a – Tinder use (yes/no) and general self-esteem

A multiple regression analysis was conducted to test whether general self-esteem differed between Tinder users and non-users, controlling for sexual self-esteem, sex, age, socioeconomic status, education, relationship status, and nationality. The adjusted model was statistically significant, explaining 19.8% of the variance in general self-esteem. Tinder use was negatively associated with general self-esteem (*B* = −0.48, *SE* = 0.24, *t* = −2.01,* p* = 0.044), with users reporting slightly lower general self-esteem than non-users. Full results are presented in Table 3.

#### H1b – Tinder use frequency and general self-esteem

A multiple regression analysis was conducted to test whether Tinder use frequency predicted general self-esteem among Tinder users, controlling for sexual self-esteem, sex, age, socioeconomic status, education, relationship status, and nationality. Again, the adjusted model was statistically significant, explaining 19.2% of the variance. Tinder use frequency was not a significant predictor of general self-esteem after adjustment (*B* = −0.03, *SE* = 0.11, *t* = −0.29, *p* = 0.774). Full results are shown in Table 3.

#### H2a – Tinder use (yes/no) and sexual self-esteem

A multiple regression analysis was conducted to test whether sexual self-esteem differed between Tinder users and non-users, controlling for general self-esteem, sex, age, socioeconomic status, education, relationship status, and nationality. The adjusted model was statistically significant, explaining 19.0% of the variance. Tinder use was a significant positive predictor of sexual self-esteem (*B* = 1.89, *SE* = 0.40, *t* = 4.78, *p* < 0.001), with users reporting higher sexual self-esteem than non-users after adjustment. Full results are presented in Table 3.

#### H2b – Tinder use frequency and sexual self-esteem

A multiple regression analysis was conducted to test whether sexual self-esteem was associated with Tinder use frequency among Tinder users, controlling for general self-esteem, sex, age, socioeconomic status, education, relationship status, and nationality. The adjusted model was statistically significant, explaining 17.4% of the variance. Tinder use frequency was not a significant predictor of sexual self-esteem after adjustment (*B* = −0.05, *SE* = 0.18,* t* = −0.27, *p* = 0.789). Full results are displayed in Table 3.

### Exploratory analyses

In exploratory analyses, we examined whether participants’ motivations for using Tinder were associated with general and sexual self-esteem. Because each of the 46 motivation items was analysed separately for both outcomes (92 correlations in total), *p*-values were adjusted using the Benjamini–Hochberg False Discovery Rate procedure to control for multiple testing. Overall, several small but statistically significant associations emerged (see Additional file 2). The strongest effects were negative correlations between general self-esteem and compensatory or emotionally driven motives, particularly items such as “I need someone to talk to” (*ρ* = −0.28), “It makes me feel less alone” (*ρ* = −0.26), “I find it easier to open up to others online than offline” (*ρ* = −0.25), “To gain more self-confidence” (*ρ* = −0.24), and “To feel more attractive” (*ρ* = −0.24). Higher endorsement of these motives was associated with lower general self-esteem, and these associations remained significant after false discovery rate correction. Associations between these same items and sexual self-esteem were present but substantially weaker. In contrast, items reflecting sexual motives (such as “I am looking for a one-night stand” and “To find someone to have sex with”) were positively associated with sexual self-esteem (*ρ* = 0.21), meaning that stronger endorsement of these motives was linked to higher sexual self-esteem.

As a robustness check, motivation items were aggregated into the six validated subscales identified by Sumter et al. [39]: Love, Casual Sex, Ease of Communication, Self-Worth Validation, Thrill of Excitement, and Trendiness. Subscale scores were computed as the mean of their respective items, with “Not Applicable” responses excluded. Internal consistency in the present sample was acceptable to good for Love (*α* = 0.85), Self-Worth Validation (*α* = 0.83), Casual Sex (*α* = 0.77), and Ease of Communication (*α* = 0.74), though poor for Thrill of Excitement (*α* = 0.62) and unacceptable for Trendiness (*α* = 0.43), consistent with Sumter et al.’s [39] original report. Spearman correlations with Benjamini–Hochberg corrected *p*-values indicated that Self-Worth Validation (*ρ* = −0.28, *p_adj* < 0.001) and Ease of Communication (*ρ* = −0.27, *p_adj* < 0.001) were the strongest negative correlates of general self-esteem, while Love, Thrill of Excitement, and Trendiness showed no significant associations. For sexual self-esteem, Ease of Communication (*ρ* = −0.17, *p_adj* < *0.0*01) and Self-Worth Validation (*ρ* = −0.08, *p_adj* = 0.003) were negatively associated, as was Love (*ρ* = −0.08, *p_adj* = 0.003), whereas Casual Sex motivation showed a positive association (*ρ* = 0.19, *p_adj* < 0.001) and Thrill of Excitement showed a small positive association (*ρ* = 0.10, *p_adj* < 0.001). These subscale-level findings converge with the item-level results reported above, further supporting the interpretation that compensatory and emotionally driven motivations are most strongly and negatively associated with general self-esteem, while motivations oriented toward casual sexual encounters and excitement were specifically associated with higher sexual self-esteem. Results are reported in Additional file 3.

## Discussion

Using a large sample of Swiss and Italian emerging adults, we tested four hypotheses examining whether Tinder use and use frequency were associated with general and sexual self-esteem. The results revealed a nuanced pattern with partial support for our hypotheses. Tinder use was associated with slightly lower general self-esteem compared to non-use (H1a), and frequency of use was not significantly associated with general self-esteem in either direction (H1b). Regarding sexual self-esteem, Tinder use (yes/no) was associated with higher sexual self-esteem (H2a), but frequency of use among users showed no significant association (H2b). These findings suggest that distinct facets of self-perception may be differentially, and, in opposing directions, associated with dating app engagement. While general self-esteem appeared slightly lower among users, sexual self-esteem appeared higher among Tinder users compared to non-users, though neither outcome was associated with the intensity of engagement among users.

Regarding H1a, the negative association between Tinder use and general self-esteem is consistent with earlier studies suggesting that dating app use is associated with lower self-esteem [38]. Several mechanisms may account for this association. Tinder’s emphasis on appearance-based evaluations and rapid partner selection may foster increased social comparison and perceived rejection, processes linked to diminished body image and global self-worth. Alternatively, individuals with lower self-esteem may be more drawn to Tinder’s lower-barrier social environment, where digital interactions may feel less threatening than face-to-face encounters. Although our cross-sectional design does not allow us to disentangle these pathways, the finding aligns with evidence that dating app users tend to report more negative body image and reduced self-esteem [38]. Contrary to H1b, frequency of Tinder use among users was not significantly associated with general self-esteem.

Regarding H2a, Tinder use was positively associated with sexual self-esteem, consistent with previous findings that Tinder users report more permissive sexual attitudes and higher sociosexuality [3, 33, 39]. One possible explanation is that Tinder facilitates sexual exploration and provides low-threshold access to potential partners, which may reinforce perceived sexual desirability and competence through positive social feedback (e.g., matches, flirtatious interactions). Alternatively, individuals with higher sexual self-esteem may be more likely to use dating apps, indicating selection effects. Contrary to H2b, frequency of use among users showed no significant association with sexual self-esteem either.

Taken together, the null frequency findings for both general (H1b) and sexual self-esteem (H2b) suggest that it is the act of engaging with Tinder, or pre-existing characteristics of those who do, rather than the intensity of use that accounts for the observed associations. This is consistent with research indicating that problematic outcomes of dating app use are more closely tied to users’ underlying motives and emotional needs than to frequency of engagement per se [45].

Across all adjusted models, general self-esteem was a significant positive predictor of sexual self-esteem, consistent with prior work suggesting that sexual self-perceptions are rooted in broader self-worth schemas [23, 30]. Being in a relationship and older age were associated with greater sexual self-esteem compared to being single and being younger, potentially reflecting the stabilising effects of a committed partnership and accumulated relational experience on sexual confidence. In contrast, having a university education showed a negative association with sexual self-esteem when comparing users to non-users (H2a), though this association was not significant among Tinder users alone (H2b). Where observed, this pattern may reflect greater self-reflective or critical engagement with sexual norms and expectations rather than diminished sexual functioning per se*.*

These exploratory findings enrich the interpretation of our main results, underscoring the importance of differentiating between motivational subtypes of Tinder use when assessing its psychological correlates. Taken together, these patterns suggest that general and sexual self-esteem respond to different aspects of the dating app experience: the swiping and evaluation dynamics may challenge broader self-perceptions, while the sexual and romantic dimensions of the app may reinforce users’ sense of sexual desirability.

These findings highlight the importance of addressing sexual self-esteem explicitly in psychoeducational and therapeutic work with emerging adults who use dating apps. Digital literacy programmes and counselling interventions could benefit from helping young people reflect on how they present themselves online, how they interpret matches and rejections, and how these experiences shape their overall sense of self-worth. Strengthening internal sources of self-esteem and fostering more reflective, autonomy-supportive app use may help mitigate the potential negative effects of dating apps on general self-worth while preserving the benefits they may offer for sexual confidence and exploration.

### Limitations and future research

Several limitations should be noted. First, the sample was predominantly composed of participants from Western, educated, industrialised, wealthy, and democratic contexts, especially university students. This restricts the generalisability of findings to broader or less digitally immersed populations. The overrepresentation of women and exclusion of non-binary individuals may have further limited external validity, overlooking potentially meaningful gender-diverse experiences of Tinder use and self-esteem. Moreover, although we collected a continuous measure of gender identity, the strong clustering of responses at the highest male/female identification values and the high correlation with sex assigned at birth required us to dichotomise the gender-identity variable for supplementary analyses. This approach, while statistically necessary to avoid multicollinearity, reduces nuance and prevents meaningful differentiation of gender-diverse identities.

Second, this study relied on self-report measures, which are subject to recall bias and socially desirable responding. It is possible that participants misrepresented or idealised their experiences, particularly regarding sexual behaviours and motivations. Moreover, because participation was voluntary, self-selection bias cannot be ruled out; individuals interested in dating apps or sexuality might have been more likely to take part.

Third, some offline behaviours were not assessed. For example, the amount of time participants spent communicating on Tinder before meeting offline, or the quality of those encounters, could provide richer insights into how online experiences translate into self-perception. In addition, the relatively high prevalence of Tinder users in our sample (64.7%) might reflect broader generational and cultural trends, as dating app use is particularly widespread among Italian and Swiss emerging adults.

Finally, the cross-sectional design precludes causal inference. Longitudinal or experimental research is needed to determine whether Tinder use leads to changes in self-esteem or whether pre-existing self-perceptions shape engagement patterns. Future studies should also examine mediating and moderating mechanisms, such as attachment style, body image, sexual satisfaction, or sociocultural norms, to better capture the pathways linking dating app behaviour with well-being. Expanding this work to include more gender-diverse and non-Western samples would enhance cultural validity and inclusivity.

Understanding the psychological mechanisms underlying Tinder use and its association with self-esteem and sexual self-esteem could inform interventions aimed at promoting healthy online dating behaviours and improving both general and sexual self-esteem.

## Conclusion

To our knowledge, this study is the first to simultaneously examine Tinder use and its associations with general and sexual self-esteem in a large sample of European emerging adults. The findings reveal a divergent pattern: Tinder users reported slightly lower general self-esteem than non-users, yet engagement with the app was associated with higher sexual self-esteem. Importantly, neither association was further amplified by frequency of use among users, suggesting that it is the act of engaging with Tinder rather than the intensity of engagement that matters for self-perceptions. These results underscore the importance of considering distinct domains of self-esteem when evaluating the psychological correlates of digital intimacy.

The impact of Tinder on self-perception likely depends on individual characteristics (including motivations, attachment style, and emotional resilience) rather than on app use alone. In this sense, Tinder may function as a social mirror that can either reinforce self-acceptance or amplify insecurities, depending on users’ internal resources and reasons for engagement. As dating apps continue to play a central role in social and romantic interaction [16], future research should examine their evolving influence on psychological well-being using longitudinal designs and more diverse samples, while applied efforts could focus on helping emerging adults engage more reflectively and draw on internal rather than external sources of self-worth.

## Supplementary Information

Additional file 1. Multiple linear regression models predicting sexual and general self-esteem from Tinder use and Tinder use frequency. This file contains all supplementary linear regression models in which gender-identification scores were used instead of sex assigned at birth. The tables report unstandardised coefficients (*B*), standard errors (*SE(B)*), standardised coefficients (*β*), t-values, and *p*-values for all predictors.

Additional file 2. Spearman correlations between 46 Tinder use motivation items and general and sexual self-esteem (Tinder users only). This file contains Spearman correlation coefficients between 46 Tinder use motivation items and general (GSE) and sexual (SSE) self-esteem. For each motivation item, the file reports correlation coefficients (ρ), *p*-values, *p*-values adjusted for multiple comparisons, and sample sizes for each correlation. The file also includes the item number corresponding to each motivation item.

Additional file 3. Spearman correlations between Tinder use motivation subscales and general and sexual self-esteem (Tinder users only). This file contains Spearman correlation coefficients between the six Tinder use motivation subscales (Love, Casual Sex, Ease of Communication, Self-Worth Validation, Thrill of Excitement, and Trendiness; [39]) and general (GSE) and sexual (SSE) self-esteem. For each subscale, the file reports correlation coefficients (ρ), *p*-values, Benjamini–Hochberg adjusted *p*-values, and sample sizes.

## Data Availability

The dataset supporting the conclusions of this article is not publicly available due to ethical restrictions related to participant confidentiality, but it is available from the corresponding author on reasonable request for secondary research purposes.

## Abbreviations

RSES: Rosenberg Self-Esteem Scale
SES: Socioeconomic status
GSE: General self-esteem
SSE: Sexual self-esteem

## Footnotes

1 The Italian translation of the Tinder Motivations Scale and the Sexual Self-Esteem Scale was produced by the first author (a native Italian speaker). The French translation was produced by a native French-speaking colleague. Both translations were reviewed for linguistic accuracy and conceptual equivalence. No formal back-translation procedure was conducted; this should be considered a limitation when interpreting findings from French- and Italian-speaking participants.

2 The “Not Applicable” option was added to the Tinder Motivations Scale to allow participants to distinguish between motivations they actively disagreed with and those they considered entirely irrelevant to their use. Responses marked as “Not Applicable” were recoded as missing and excluded pairwise from analyses.

3 The “Not Applicable” option was added to the RSES and Sexual Self-Esteem Scale to allow participants to skip items they felt unable to respond to meaningfully (e.g., items about sexual experience or self-worth in contexts they had not encountered). These responses were treated as missing and handled via pairwise deletion.

4 Italian, French, and German versions of the Sexual Self-Esteem Scale were produced through forward translation by the first author and reviewed by bilingual colleagues. No formal back-translation procedure was conducted; this should be considered a limitation when interpreting findings from non-English-speaking participants.

5 To assess the robustness of findings when using self-reported gender identity rather than sex assigned at birth, all four regression models were re-estimated using a dichotomised gender-identity variable (male vs. female identification). Among participants assigned female at birth, 90.8% reported strongly identifying as women, and among those assigned male at birth, 88.9% reported strongly identifying as men (score = 5 on a 0–5 scale). Because gender identity and sex assigned at birth were highly correlated, including both simultaneously would have introduced multicollinearity. The direction and significance of results were consistent across all models, with the exception that the association between Tinder use and general self-esteem (H1a) was in the same negative direction but did not reach statistical significance in the gender-identity model. All results are reported for gender identification in Additional file 1.

## References

[1] Abramova O, Baumann A, Krasnova H, Buxmann P. Gender differences in online dating: what do we know so far? A systematic literature review. In: 2016 49th Hawaii International Conference on System Sciences (HICSS). 2016. p. 3858–3867. 10.1109/hicss.2016.481.

[2] Barrada JR, Castro Á. Tinder users: sociodemographic, psychological, and psychosexual characteristics. Int J Environ Res Public Health. 2020;17(21):8047. 10.3390/ijerph17218047. 33142900 10.3390/ijerph17218047 PMC7662763

[3] Bonilla-Zorita G, Griffiths MD, Kuss DJ. Online dating and problematic use: a systematic review. Int J Ment Heal Addict. 2021;19(6):2245–78. 10.1007/s11469-020-00318-9.

[4] Breslow AS, Sandil R, Brewster ME, Parent MC, Chan A, Yucel A, et al. Adonis on the apps: online objectification, self-esteem, and sexual minority men. Psychology of Men & Masculinities. 2020;21(1):25–35. 10.1037/men0000202. 38827385 10.1037/men0000202 PMC11142472

[5] Carpenter CJ, McEwan B. The players of micro-dating: Individual and gender differences in goal orientations toward micro-dating apps. First Monday. 2016. 10.5210/fm.v21i5.6187.

[6] Ciocca G, Robilotta A, Fontanesi L, Sansone A, D’Antuono L, Limoncin E, et al. Sexological aspects related to tinder use: a comprehensive review of the literature. Sex Med Rev. 2020;8(3):367–78. 10.1016/j.sxmr.2019.12.004. 32089467 10.1016/j.sxmr.2019.12.004

[7] Comtois D. Tools to quickly and neatly summarize data [R package summarytools] (Version 1.0.1) [R]. 2022. https://www.semanticscholar.org/paper/Tools-to-Quickly-and-Neatly-Summarize-Data-%5BR-Comtois/b3221febe9ae549c6366f9c1f3779f5f87503aa9.

[8] Dapp LC, Orth U. Rank-order stability of domain-specific self-esteem: a meta-analysis. J Pers Soc Psychol. 2024;127(2):432–54. 10.1037/pspp0000497. 38451709 10.1037/pspp0000497

[9] Diener E, Diener M. Cross-Cultural correlates of life satisfaction and self-esteem. In: Diener E, editor. Culture and well-being, vol. 38. Springer Netherlands; 2009. p. 71–91. 10.1007/978-90-481-2352-0_4.

[10] Doyle Zeanah P, Schwarz JC. Reliability and validity of the sexual self-esteem inventory for women. Assessment. 1996;3(1):1–15. 10.1177/107319119600300101.

[11] Freire T, Tavares D. Influência da autoestima, da regulação emocional e do gênero no bem-estar subjetivo e psicológico de adolescentes. Arch Clin Psychiatry (São Paulo). 2011;38(5):184–8. 10.1590/S0101-60832011000500003.

[12] Gudelunas D. There’s an app for that: the uses and gratifications of online social networks for gay men. Sex Cult. 2012;16(4):347–65. 10.1007/s12119-012-9127-4.

[13] Hashim N, Bolong J, Razak MNF. Viewers’ choice: discrete choice elements of viewer’s in portal television usage and preferred types of programs watched frequency in achieving gratifications. Int J Acad Res Bus Soc Sci. 2021;11(8):1867–89. 10.6007/IJARBSS/v11-i8/10689.

[14] Her YC, Timmermans E. Tinder blue, mental flu? Exploring the associations between Tinder use and well-being. Inf Commun Soc. 2021;24(9):1303–19. 10.1080/1369118X.2020.1764606.

[15] Iannaccone M, D’Olimpio F, Cella S, Cotrufo P. Self-esteem, body shame and eating disorder risk in obese and normal weight adolescents: a mediation model. Eat Behav. 2016;21:80–3. 10.1016/j.eatbeh.2015.12.010. 26789834 10.1016/j.eatbeh.2015.12.010

[16] Lee SS. Permanent connectedness and its impact on news sharing. New Media Soc. 2023;25(11):3117–36. 10.1177/14614448211063466.

[17] Maas MK, Lefkowitz ES. Sexual esteem in emerging adulthood: associations with sexual behavior, contraception use, and romantic relationships. J Sex Res. 2015;52(7):795–806. 10.1080/00224499.2014.945112. 25210789 10.1080/00224499.2014.945112 PMC4362922

[18] Mastro S, Zimmer-Gembeck MJ. Let’s talk openly about sex: sexual communication, self-esteem and efficacy as correlates of sexual well-being. Eur J Dev Psychol. 2015;12(5):579–98. 10.1080/17405629.2015.1054373.

[19] May A, Johnston KL. Ego-centred and partner/activity-focused sexual satisfaction: the role of self-esteem and sexual assertiveness in cisgender heterosexual women. Sex Roles. 2022;86(3–4):179–88. 10.1007/s11199-021-01258-x.

[20] Menon D. Tinder versus bumble: how do life position indicators and usage motivations predict dating? Sex Cult. 2024;28(6):2797–824. 10.1007/s12119-024-10257-5.

[21] Monteiro RP, Coelho GLH, Hanel PHP, de Medeiros ED, da Silva PDG. The efficient assessment of self-esteem: proposing the brief Rosenberg Self-Esteem Scale. Appl Res Qual Life. 2022;17(2):931–47. 10.1007/s11482-021-09936-4.

[22] Nader K. The gamification of dating online. Theoria. 2025;91(3):e12549. 10.1111/theo.12549.

[23] Oattes MK, Offman A. Global self-esteem and sexual self-esteem as predictors of sexual communication in intimate relationships. Can J Hum Sex. 2007;16. https://openurl.ebsco.com/EPDB%3Agcd%3A8%3A349512/detailv2?sid=ebsco%3Aplink%3Ascholar&id=ebsco%3Agcd%3A31123761&crl=c&link_origin=none.

[24] Orosz G, Benyó M, Berkes B, Nikoletti E, Gál É, Tóth-Király I, et al. The personality, motivational, and need-based background of problematic Tinder use. J Behav Addict. 2018;7(2):301–16. 10.1556/2006.7.2018.21. 29642722 10.1556/2006.7.2018.21 PMC6174578

[25] Orth U, Dapp LC, Erol RY, Krauss S, Luciano EC. Development of domain-specific self-evaluations: a meta-analysis of longitudinal studies. J Pers Soc Psychol. 2021;120(1):145–72. 10.1037/pspp0000378. 33252972 10.1037/pspp0000378

[26] R Core Team. R: a language and environment for statistical computing (version 4.4.2) [Computer software]. R Foundation for Statistical Computing; 2024. https://www.R-project.org.

[27] Ranzini G, Lutz C. Love at first swipe? Explaining Tinder self-presentation and motives. Mobile Media Commun. 2017;5(1):80–101. 10.1177/2050157916664559.

[28] Rosenberg M. Society and the adolescent self-image. Princeton University Press; 1965. https://www.jstor.org/stable/j.ctt183pjjh.

[29] Safshekan S, Khalesi ZB. Factors affecting sexual-self-esteem among Iranian women. Eur J Obstet Gynecol Reprod Biol X. 2024;21:100284. 10.1016/j.eurox.2024.100284. 38323102 10.1016/j.eurox.2024.100284 PMC10845242

[30] Sakaluk JK, Kim J, Campbell E, Baxter A, Impett EA. Self-esteem and sexual health: a multilevel meta-analytic review. Health Psychol Rev. 2020;14(2):269–93. 10.1080/17437199.2019.1625281. 31163109 10.1080/17437199.2019.1625281

[31] Salmela-Aro K, Nurmi J-E. Self-esteem during university studies predicts career characteristics 10 years later. J Vocat Behav. 2007;70(3):463–77. 10.1016/j.jvb.2007.01.006.

[32] Segabinazi JD, Zortea M, Zanon C, Bandeira DR, Giacomoni CH, Hutz CS. Positive and negative affect scale for adolescents: Adaptation, standardization and validity evidence. Aval Psicol. 2012;11(1):1–12.

[33] Sevi B, Aral T, Eskenazi T. Exploring the hook-up app: low sexual disgust and high sociosexuality predict motivation to use Tinder for casual sex. Pers Individ Differ. 2018;133:17–20. 10.1016/j.paid.2017.04.053.

[34] Snell WE, Papini DR. The sexuality scale: an instrument to measure sexual-esteem, sexual-depression, and sexual-preoccupation. J Sex Res. 1989;26(2):256–63. 10.1080/00224498909551510.

[35] Sowislo JF, Orth U. Does low self-esteem predict depression and anxiety? A meta-analysis of longitudinal studies. Psychol Bull. 2013;139(1):213–40. 10.1037/a0028931. 22730921 10.1037/a0028931

[36] Steiger AE, Allemand M, Robins RW, Fend HA. Low and decreasing self-esteem during adolescence predict adult depression two decades later. J Pers Soc Psychol. 2014;106(2):325–38. 10.1037/a0035133. 24467425 10.1037/a0035133

[37] Strübel J. The most swipeable you: experiences and self-perception of Tinder users. J Am Cult. 2023;46(1):44–54. 10.1111/jacc.13428.

[38] Strubel J, Petrie TA. Love me Tinder: body image and psychosocial functioning among men and women. Body Image. 2017;21:34–8. 10.1016/j.bodyim.2017.02.006. 28285177 10.1016/j.bodyim.2017.02.006

[39] Sumter SR, Vandenbosch L, Ligtenberg L. Love me Tinder: untangling emerging adults’ motivations for using the dating application Tinder. Telematics Inform. 2017;34(1):67–78. 10.1016/j.tele.2016.04.009.

[40] Szcześniak M, Bajkowska I, Czaprowska A, Sileńska A. Adolescents’ self-esteem and life satisfaction: communication with peers as a mediator. Int J Environ Res Public Health. 2022;19(7):3777. 10.3390/ijerph19073777. 35409459 10.3390/ijerph19073777 PMC8997743

[41] Thomas MF, Binder A, Matthes J. The agony of partner choice: The effect of excessive partner availability on fear of being single, self-esteem, and partner choice overload. Comput Hum Behav. 2022;126:106977. 10.1016/j.chb.2021.106977.

[42] Thomas MF, Binder A, Stevic A, Matthes J. 99 + matches but a spark ain’t one: adverse psychological effects of excessive swiping on dating apps. Telematics Inform. 2023;78:101949. 10.1016/j.tele.2023.101949.

[43] Timmermans E, De Caluwé E. Development and validation of the Tinder Motives Scale (TMS). Comput Hum Behav. 2017;70:341–50. 10.1016/j.chb.2017.01.028.

[44] Van De Wiele C, Tong ST. Breaking boundaries: the uses & gratifications of grindr. In: Proceedings of the 2014 ACM International Joint Conference on Pervasive and Ubiquitous Computing. 2014. p. 619–630. 10.1145/2632048.2636070.

[45] Vera Cruz G, Aboujaoude E, Rochat L, Bianchi-Demicheli F, Khazaal Y. Online dating: predictors of problematic tinder use. BMC Psychol. 2024;12(1). 10.1186/s40359-024-01566-3. 10.1186/s40359-024-01566-3 PMC10905798 38424651

[46] Von Soest T, Wichstrøm L, Kvalem IL. The development of global and domain-specific self-esteem from age 13 to 31. J Pers Soc Psychol. 2016;110(4):592–608. 10.1037/pspp0000060. 26167796 10.1037/pspp0000060

[47] Wickham H. ggplot2: elegant graphics for data analysis [Computer software]. Springer-Verlag; 2016. https://ggplot2.tidyverse.org/book/.

[48] Wickham H, Averick M, Bryan J, Chang W, McGowan L, François R, et al. Welcome to the Tidyverse. J Open Source Softw. 2019;4(43):1686. 10.21105/joss.01686.

[49] Wickham H, Miller E, Smith D. haven: import and export “SPSS”, “Stata” and “SAS” files. (Version 2.5.4) [R]. 2023. https://CRAN.R-project.org/package=haven.

[50] Wiederman MW, Allgeier ER. The measurement of sexual-esteem: investigation of Snell and Papini′s (1989) sexuality scale. J Res Pers. 1993;27(1):88–102. 10.1006/jrpe.1993.1006.

[51] Zell E, Johansson JS. The association of self-esteem with health and well-being: a quantitative synthesis of 40 meta-analyses. Soc Psychol Pers Sci. 2025;16(4):412–21. 10.1177/19485506241229308.

